# Abscess of the Brain

**Published:** 1907-01

**Authors:** 


### ABSCESS OF THE BRAIN.

---

Wm. G. Spiller (*Pennsylvania Medical Journal,* October, 1906) says that the diagnosis of cerebral abscess depends chiefly upon the signs of some more or less rapidly developing lesions of the brain, with the discovery of a purulent process somewhere in the body, or of a wound of the head. Abscess of the brain without any evidence of pus elsewhere may be extremely difficult to diagnose. The cerebral signs are much the same as those of tumor of the brain. The choked disk may occur in either condition, but it is more likely to be intense in tumor and to occur earlier; the writer has observed choked disk of high grade in abscess. The temperature and pulse are likely to be subnormal in abscess, but they may be normal or even above normal, especially if the abscess is complicated by meningitis. We should not depend too much on the condition of the pulse and temperature. The temperature is more likely to rise in the terminal stage of abscess, but a rise may occur at any period and be caused by the primary suppurative process, as a superficial wound or purulent pulmonary disease. The stupor, in his experience, is usually greater in advanced stages of abscess than in tumor, and yet an abscess may be latent or nearly latent for a long time, and stupor may be pronounced with brain tumor. It is not uncommon to find slow cerebration preceding stupor in either condition. In many cases the diagnosis must be in doubt, but given a case of long-standing purulent otitis media, and recently developed signs of disease of the temporal lobe or cerebellum, the diagnosis of abscess becomes more positive. High leucocytosis speaks in favor of abscess or purulent meningitis in contrast with tumor. Where the cerebral symptoms appear after a purulent pulmonary process the diagnosis between tumor and abscess of the brain is difficult. Virchow is said to have been the first to call attention to the frequency of abscess following a purulent affection of the lungs. Still more difficult is the determination as to whether purulent meningitis or brain abscess is present, and often the two conditions are associated in the same person.

The diagnosis is important, because less can be hoped for from operation if purulent meningitis develops, whereas operation is demanded if the condition is one of abscess. Lumbar puncture is of great value, as pus in the cerebrospinal fluid obtained by this means points to purulent meningitis; but purulent meningitis may exist althouhg pus in the spinal fluid is not obtained. The presence of mononuclear or polynuclear cells in the cerebrospinal fluid is also of diagonistic value. Kernig's sign is of value as indicating meningitis; but is not pathognomonic and may occur with abscess. Rigidity of the neck is more likely to occur in meningitis, but may occur with abscess. Where abscess is associated with purulent meningitis the prognosis is extremely grave.—*Medical Age.*

